# The Spread of Exhaled Air and Aerosols during Physical Exercise

**DOI:** 10.3390/jcm12041300

**Published:** 2023-02-06

**Authors:** Hayder Alsaad, Gereon Schälte, Mario Schneeweiß, Lia Becher, Moritz Pollack, Amayu Wakoya Gena, Marcel Schweiker, Maria Hartmann, Conrad Voelker, Rolf Rossaint, Matthias Irrgang

**Affiliations:** 1 Department of Building Physics, Faculty of Civil Engineering, Bauhaus-University Weimar, 99423 Weimar, Germany; 2 Department of Anesthesiology, Medical Faculty, University Hospital RWTH Aachen, 52074 Aachen, Germany; 3 Healthy Living Spaces Lab, Institute for Occupational, Social, and Environmental Medicine, Medical Faculty, RWTH Aachen University, 52074 Aachen, Germany

**Keywords:** sport, training, cycle ergometer, schlieren imaging, particles concentration, user satisfaction

## Abstract

Physical exercise demonstrates a special case of aerosol emission due to its associated elevated breathing rate. This can lead to a faster spread of airborne viruses and respiratory diseases. Therefore, this study investigates cross-infection risk during training. Twelve human subjects exercised on a cycle ergometer under three mask scenarios: no mask, surgical mask, and FFP2 mask. The emitted aerosols were measured in a grey room with a measurement setup equipped with an optical particle sensor. The spread of expired air was qualitatively and quantitatively assessed using schlieren imaging. Moreover, user satisfaction surveys were conducted to evaluate the comfort of wearing face masks during training. The results indicated that both surgical and FFP2 masks significantly reduced particles emission with a reduction efficiency of 87.1% and 91.3% of all particle sizes, respectively. However, compared to surgical masks, FFP2 masks provided a nearly tenfold greater reduction of the particle size range with long residence time in the air (0.3–0.5 μm). Furthermore, the investigated masks reduced exhalation spreading distances to less than 0.15 m and 0.1 m in the case of the surgical mask and FFP2 mask, respectively. User satisfaction solely differed with respect to perceived dyspnea between no mask and FFP2 mask conditions.

## 1. Introduction

The restrictions associated with COVID-19 were lifted between the infection waves and fitness studios opened without clear information about the spread of exhaled aerosols and droplets in such venues. This can increase the cross-infection rates among the users of these rooms, especially with the lack of safety guidance specific for dynamic activities involving higher emission rates of potentially infectious aerosols and droplets, which can cause upper and lower respiratory infections [1].

The exhaled droplets have a polydisperse size distribution between 0.01–500 µm [2]. In this context, droplets can be classified as large droplets with a size of >100 μm, medium droplets with a size of 5–100 μm, and small droplets (aerosols) with a size of <5 μm [3]. The small particles (with a threshold of 5 µm or less) are commonly called “droplet nuclei.” Since they remain almost indefinitely airborne in enclosed rooms and are prone in the lower respiratory tract of humans [4,5,6], small particles are predominantly responsible for airborne transmitted diseases.

Studies characterizing exhaled droplets focus mainly on measuring the number as well as the size of the initial droplets, as this is the most important parameter to determine how quickly they accumulate on a surface or whether they are carried on by the airflow. Morawska et al. [7] were able to assign specific particle modal values to different exhalation activities. The number of emitted particles differs strongly between different test subjects. Therefore, Hartmann et al. [8] describe low, high, and super emitters. Furthermore, studies suggest that droplet size is associated with the aerosol composition as well as the probability of containing pathogens [9,10]. Moreover, the biology of the pathogen, such as lipid- or non-lipid enveloped, and environmental factors such as air temperature and humidity are crucial for its airborne survival and infectivity [11].

With the severe acute respiratory syndrome (SARS) outbreak in 2002, caused by a coronavirus (SARS-CoV), the behavior of and protection from airborne infectious pathogens became and still are subject of current research [12]. It can be demonstrated, without contradiction, that wearing a protective face mask alters the spread of exhaled air, sneezing, and coughing. Face masks provide efficient protection against transmissions, presupposed high-quality masks with a specimen adequate class of protection are worn properly [13,14]. In early 2020, the first recommendations and guidelines for preventing transmission and infection could be published due to previous research on SARS outbreak [15]. Since the occurrence of COVID-19, it was hypothesized that the new coronavirus 2 was transmitted via infectious aerosols [16], recommendations for self-protection evolved from social distancing, wearing protective gowns and/or other face covering including filtrating masks with different protection classes depending on the intensity of contact [17,18].

The general physical behavior of aerosols in enclosed rooms and thus various opportunities to decrease the risk of transmission by optimizing ventilation and sanitizing the air have been examined in the literature [3,19]. Yet, the effect of physical stress, e.g., in gyms or medical facilities, on aerosol generation and spreading is still a subject of current research and has to be investigated in detail [20,21,22]. Parallel to the assessment of aerosol spreading and protective masks during physical stress, user satisfaction should also be investigated, as the use of face masks can affect user comfort and exertion. In their review, Zheng et al. [23] found significantly increased Borg ratings of perceived exertion (RPE) for surgical masks, but not for filtering facepiece class 2 (FFP2) or cloth masks. In addition, reported perception of dyspnea (RPD), fatigue level, and thermal sensation increased with face mask usage. Ipek et al. [24] compared using surgical and N95 masks among healthcare workers and detected respiratory alkalosis and hypocarbia after the use of N95 masks. However, the findings are not conclusive. For example, Yoshihara et al. [25] looked at the effect of four different face masks (surgical, N95, gaiter, and sport) on objective and subjective measures when walking and jogging in temperatures above 32 °C. After 30 and 60 min exposures, only overall breathing discomfort was higher in trials with face masks compared to the control condition. Other measures (RPE, thermal sensation, thirst sensation, and fatigue level) did not differ significantly. Therefore, adding more insights into the effect of using face masks during exercise on subjective measures is necessary.

The present study evaluates the characteristics of emitted aerosols and the spread of exhaled air during a standardized physical exercise protocol in a laboratory setup. The goal of the investigations is to assess the efficacy of reducing infectious transmission using widely-available face masks during training. The present study hypothesizes that wearing any kind of medical face mask during physical exercise can establish significant protection against the spread of exhaled airborne particles, thus providing effective protection against airborne diseases. The investigations included three main parts: (1) aerosol measurements to assess the reduction of the released particles when face masks are used, (2) airflow visualization to assess the distribution and spreading distance of the exhaled air when face masks are used, and (3) user satisfaction surveys to assess the acceptance of wearing face masks during exercise.

For aerosol measurements, a wide range of different measurement principles can be applied. While solid impactors and droplet separators were primarily used in former studies, current studies use optical and aerodynamic particle sizers. In individual cases, liquid impactors, differential mobility analyzers, low-pressure electrical impactors, and condensation particle counters were applied as well. An overview of the application of these various measurement techniques to characterize breathing air is given by Merghani et al. [26]. In the present study, the aerosols measurements were conducted using an optical particle sensor. For airflow visualizations, the literature also reports multiple techniques such as particle image velocimetry (PIV) [27], particle streak tracking (PST) [28], smoke visualizations [29], and schlieren imaging [30]. The latter has been utilized in this study due to the flexibility and high resolution of this approach that provides two-dimensional visualizations of the flow field with virtually no disturbance to the flow. Therefore, this visualization method has been frequently used in recent literature to evaluate indoor air quality topics, such as the spread of air from face masks [31], from nasal high-flow therapy [32], from music instruments [33], and ventilation strategies [34]. Detailed information about the utilized schlieren imaging system is presented in Section 2.2. For user satisfaction assessments, questionnaires were distributed after the airflow visualization of each investigated scenario. Detailed information about the user satisfaction surveys is presented in Section 2.3.

## 2. Materials and Methods

Twelve healthy adult test subjects (six females and six males) participated in the study with an age ranging from 24 to 54 years with an average of 34.3 years and a median of 27.5 years. The test subjects had diverse physical conditions with a general tendency towards training at least 2 h a week. The body mass index (BMI) of the subjects ranged from 19.7 to 23.8 kg/m^2^ with an average of 22.3 kg/m^2^ and a median of 22.7 kg/m^2^. The subjects had typical sports clothing (sports tops or T-shirts; sports shorts or trousers). All subjects signed a consent to voluntarily participate in the study and an approval for conducting this study was obtained from the Ethical Board of the Faculty of Medicine, RWTH Aachen University (protocol code EK 312/21). All subjects were double-vaccinated against COVID-19 at the time of the study (October 2021). Moreover, a SARS-CoV-2 rapid antigen test was performed under the supervision of a medical professional for all test subjects and participating researchers at the beginning of each investigation day. Furthermore, the test setups were disinfected after each measurement set; hand disinfectant was available for all subjects and researchers throughout the investigations.

All the investigations were conducted using a stationary bike (a cycle ergometer) as an exemplary device that is often used in gyms and fitness studios. Each subject exercised on the cycle ergometer under three exercise conditions: with no mask, with a disposable medical facemask (surgical mask), and with an FFP2 mask. Each scenario was investigated twice: for measuring the emitted aerosols (Section 2.1) and for visualizing the expired air and conducting the user-satisfaction surveys (Section 2.2 and Section 2.3). Moreover, the subjects completed an additional training set for warmup before the beginning of the investigations. Thus, each test subject trained for a total of seven times on the cycle ergometer with a duration of 1–4 min per scenario. The pulse rate and blood oxygen saturation of the test subjects were constantly monitored throughout the experiments using a pulse oximeter (Masimo Radical 7, Masimo Cooperation, Irvine, CA, USA). Each investigated scenario was conducted by asking the test subjects to train at their own pace until reaching 75% of the maximum heart rate, which was determined by the modified Karvonen equation (HR_max_ = 206.9 − (0.67 × age)). For the aerosol measurements, the test subjects were asked to train for an additional 30 s after reaching 75% of the maximum heart rate in order to measure an average value for the emitted aerosols.

The indoor conditions were constantly measured and monitored during all investigations. The indoor air temperature was measured at 0.1, 1.1, and 1.7 m above the floor using negative temperature coefficient thermistors (NTC) with an accuracy of ±0.2 K. Relative humidity was monitored at 1.1 m above the floor using a capacitive sensor with an accuracy of ±2% RH. Additionally, during the aerosol measurements, the CO_2_ concentration in the room was constantly monitored at 1.1 m height using a non-dispersive infrared digital sensor with an accuracy of ±(50 ppm +3% of measured value). The indoor environmental parameters were measured using Almemo sensors system (Ahlborn GmbH, Holzkirchen, Germany).

### 2.1. Aerosols Measurements

**Figure 1:**
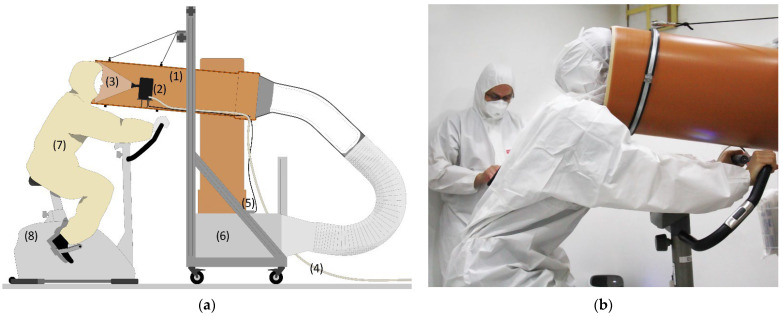
(**a**) The aerosol measurement setup in the lab: (1) PVC pipe, (2) optical particle sensor, (3) collection funnel, (4) plastic tubing to the vacuum pump, (5) data logger, (6) box with a HEPA filter, (7) test subject, and (8) cycle ergometer. (**b**) A test subject while measuring the aerosols emission rate.

The aerosol emission measurements were conducted in a grey room at the Bauhaus-University Weimar; this room is a cuboid climate chamber with the dimensions of 3 × 3 × 2.4 m (~22 m^3^) situated in a laboratory hall to keep it isolated from the outdoor environment. The chamber utilizes a ventilation system with two air inlets equipped with high-efficiency particulate absorbing (HEPA) filters to introduce clean, tempered outdoor air at an adjustable rate into the chamber. Moreover, the temperature in the chamber is also controllable by setting the temperature of each interior surface separately, which are tempered by water-bearing capillary tubes placed under the finishing layer (tiles for the floor and gypsum plaster for the walls and ceiling). An aerosol measurement setup was positioned at the center of the chamber. As shown in Figure 1a, this setup consisted of a polyvinyl chloride (PVC) pipe with an inside diameter of 29.5 cm connected to an air filter via a corrugated aluminum tube. The pipe was mounted on a structure equipped with pulleys that allowed adjustment of the height and inclination of the pipe. A 4-channel optical particle sensor (Airnet-II, PMS Inc., Boulder, CO, USA) was placed inside the pipe; the inlet of this sensor was connected to a funnel to allow capture of as many exhaled particles as possible. The implemented particle sensor can detect particles with a size range of 0.3–0.5, 0.5–1.0, 1.0–5.0, and >5.0 µm at an ISO 21501-4 [35] compliant counting efficiency of 100% ± 10% at 1.5 to 2.0 times channel one size and 50% ± 20% for the most sensitive channel. The sensor detects particles at a flow rate of 28.3 L/min (±15%), which was generated through a vacuum pump with an absolute pressure of about 400 mbar. The vacuum pump was connected to the particle sensor via Ø10 mm plastic tubing; the vacuum pump was positioned outside the chamber and its air outlet was extended to the outside to eject the drawn expired particles.

To keep the particle concentrations as low as possible in the chamber, only two persons were present inside the chamber during the experiments: the test subject and a medical professional to supervise the experiment and determine the maximum heart rate. The measurement devices and chamber setups were controlled from outside the chamber. To exclude the exogenously generated particles (e.g., from the hair and clothes) from the measurements, the test subjects and the accompanying medical professional were dressed in a hooded suit covering their whole body, neck, and hair (Figure 1b). To measure the expired aerosols, the test subjects trained on the cycle ergometer while inserting their head into the PVC pipe and facing the inlet funnel of the particle sensor. Before each measurement set, i.e., before investigating each mask condition, the particle concentration in the room was constantly monitored. Once a stable concentration was reached, the test subject was signaled to sit on the cycle ergometer and start training. To accelerate reaching a stable background concentration, the ventilation system of the chamber was set to the highest possible level (200 m^3^/h). Moreover, two portable air cleaners were placed in the chamber. A third air cleaner was positioned in the laboratory in which the chamber is situated to reduce the particle concentration peaks when the chamber door is open to let the test subject in or out. To reduce the waiting time between the three investigated exercise conditions (no mask, surgical mask, and FFP2 mask), the measurements were conducted in the following order: FFP2 mask, surgical mask, and then with no mask. This is because the initial measurements showed only a slight impact on the background particle concentration in the chamber during the FFP2 mask case, while the no mask case caused a spike in the background concentration that required a long time to flatten (Figure 3, Section 3.1). To remain consistent with the other sections of the study, the results of the aerosol measurements were, nevertheless, presented in the order of no mask (reference case), surgical mask, and FFP2 mask.

All aerosol measurements were conducted under similar climatic conditions with an average room air temperature of 21.41 °C (measured at H = 1.1 m). Since the chamber was tempered by setting all surfaces to the same temperature, the vertical temperature stratification was extremely low and laid within or near the ±0.2 K accuracy range of the implemented sensors ($\bar{\theta }$_1.1−0.1_ = 0.11 K; $\bar{\theta }$_1.7−0.1_ = 0.24 K). The average relative humidity during the measurements was 46.3% and the CO_2_ concentration in the chamber never exceeded 1212 ppm (with an average of 1007 ppm). This concentration is barely above the widely-considered “harmless” threshold of 1000 ppm [36]. However, due to the short-term exposure, it is unlikely to have negative effects on the test subjects.

### 2.2. Airflow Visualization

**Figure 2:**
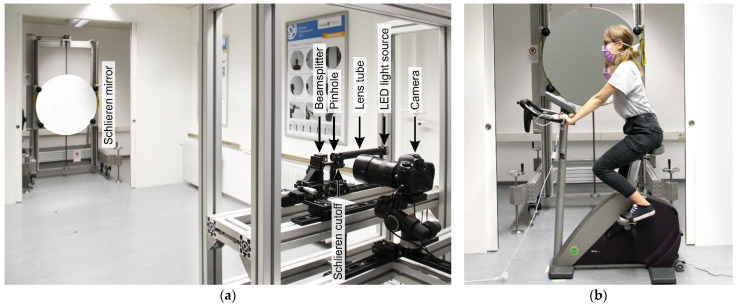
(**a**) The setup of the single-mirror coincident schlieren imaging system; (**b**) A test subject training on the cycle ergometer in front of the schlieren mirror.

The airflow visualizations were conducted using the schlieren imaging system at the Bauhaus-University Weimar. The main component of this system is the schlieren mirror, which is a concave spherical mirror with astronomical quality with a surface accuracy of λ/9.75 @ 633 nm (where λ is the wavelength of light [nm]). The mirror has a diameter of 1 m, a focal length of 3001.5 mm, and a curvature radius of 6003 mm. In addition to the mirror, the system consists of an optical setup including a light-emitting diode (LED) light source, a condenser lens, a beam splitter, a schlieren cutoff, and a high-resolution digital camera (Canon EOS 5DS R, Canon Inc., Tokyo, Japan) with 50.6 megapixels image size, full HD video, 50 fps frame rate, and a 135 mm focal-length lens with a minimum aperture of f/2.0 (Figure 2a).

This imaging system relies on the refraction of light rays when they pass through fluids with a density gradient (i.e., differences in temperature or pressure in the medium). When a light ray emitted from the LED reaches the mirror, it returns along the coincident path where it crosses the test object, which is inducing the density gradient. When passing through the density gradients, some rays are refracted and a portion of them is blocked by the schlieren cutoff. This creates shadows in the image, which constitutes the foreground of the schlieren image. The returning light rays that are not blocked by the schlieren cutoff pass to the camera sensor and brighten the image screen; this constitutes the background of the schlieren image. Further information about the principle of this schlieren imaging system can be found in Gena et al. [37].

The flow visualization of the expired air was conducted by placing a cycle ergometer at about 1 m in front of the schlieren mirror. The test subjects were asked to exercise on the cycle ergometer while looking forward at a predefined fixed point at their eye level to have a relatively uniform position and direction of the mouth and the nose during all airflow visualizations (Figure 2b). This was necessary to allow comparison of the generated exhaled cloud among the test subjects. Unlike the aerosol measurements, the order of the investigated exercise conditions (no mask, surgical mask, and FFP2 mask) was randomized during the flow visualizations to vary the implementation of the masks. This was necessary as the user satisfaction surveys were handed to the test subjects after the visualization of each exercise scenario in front of the mirror. Further details about these surveys are presented in Section 2.3.

The visualizations were conducted in a laboratory room with an air volume of ~198 m^3^. The room has no active cooling or ventilation systems. However, the room was regularly ventilated by opening a window between the investigated exercise scenarios to ensure a minimum infection risk among the test subjects. The heating system of the room (wall-mounted hot-water radiators) was switched off to avoid affecting the visualized flow through the convective flow generated by the radiators. The average room temperature measured at the height of 1.1 m was 21.58 °C during all the investigated scenarios with a slight temperature stratification of $\bar{\theta }$_1.1−0.1_ = 0.36 K and $\bar{\theta }$_1.7−0.1_ = 0.38 K. The relative humidity in the room had an average of 46.5% during all investigations.

The flow visualizations were recorded as separate video sequences for each test subject and each tested mask condition. To analyze the spreading distance of the ejected airflow, a MATLAB code was implemented to explode the schlieren videos to single frames based on the 50 Hz frequency at which the schlieren videos were recorded. The frames with fully visible structures of the exhaled air were manually sorted out. A CAD software was then used to calibrate the scale of the frames and, subsequently, measure the extent of the wavefront of the exhaled cloud.

### 2.3. User Satisfaction Surveys

As mentioned above, immediately after the airflow visualization of each exercise condition, participants were asked to fill out two questionnaires: (a) The RPE scale [38] and RPD [39], and (b) a general state questionnaire assessing 7 items as follows: (1) thirst sensation, (2) thermal sensation, and (3) fatigue level (all three from Yoshihara et al. [25]), as well as four specific complaints: (4) headache, (5) dizziness, (6) facial sweating, and (7) coughing (all four taken from İpek et al. [24]). While Ipek et al. [24] used 18 complaints, only four were chosen here, which resulted in a statistically significant difference in their study and were not redundant to other information collected to minimize the length of this repeated questionnaire.

In addition, a background questionnaire was provided once including

- Demographics (gender, age, weight, height, health status, and frequency of training);
- A 32-item Face Mask Perceptions Scale (FMPS) [40] (mean 2.15 ± 0.6, median 1.9, min 1.4, max 3.1);
- The 5-item Environmental Worry Scale according to VDI 3883 Part 1, Appendix A [41] (mean 11.3 ± 2.2, median 11, min 8, max 15).

Due to the limited sample size, only the main targets were analyzed using the complete sample and no further analysis of influences such as demographics or other background variables could be conducted. The rating scales were analyzed using one-way repeated measures ANOVA after checking for outliers, normality assumptions, and assumptions of sphericity. Multiple pairwise paired *t*-tests between the levels of the within-subject factor condition were performed using Bonferroni correction for the post-hoc tests when the ANOVA showed a significant effect. All statistical analyses were performed using the statistical software R.

## 3. Results

### 3.1. Aerosols Measurements

Since the measurements were conducted in a grey room, not in a clean room, a significant background aerosol concentration was present throughout the investigations. Therefore, to ascertain the emitted exhaled particles from the subjects, the background concentration had to be calculated for each test subject for each investigated mask condition. The mean background concentration was determined from the measured values for a duration of 30 s before the subject started training. Since the subjects took about 20 s to position themselves on the cycle ergometer before each measurement scenario, the background concentration was determined from the values measured prior to this 20 s transitional duration to avoid possible measurement inaccuracies due to the movement of the subjects toward the cycle ergometer. Following the calculation of the background concentrations, they were subtracted from the measured values to quantify the emitted particles during the evaluated mask conditions; any resulting negative values were set to zero.

**Figure 3:**
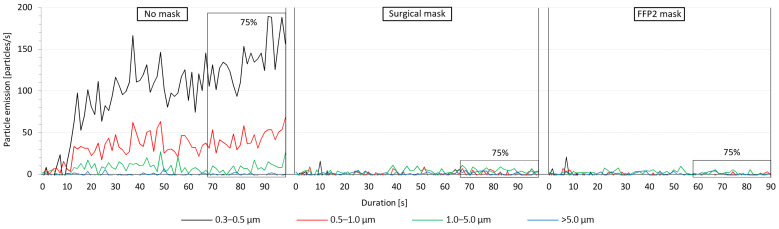
Exemplary particle emission curves from a male test subject during the tested mask conditions. The 75%-markings show the 30 s period after 75% of the maximum heart rate was reached.

Figure 3 presents an example of the measured particles emitted from a 28-year-old male test subject. The emitted particles during the three investigated mask conditions cases are illustrated in the three sections of the diagram, each starting at the time the subject started training (*t* = 0 s). The boxes marked with 75% refer to the training time after reaching 75% of the maximum heart rate, which continued for 30 s before stopping the exercise. This 30 s time span was consistent in all the conducted aerosol measurements and was used to determine the average released particles. Figure 3 indicates a remarkable difference in the distribution of the emitted particles among the tested conditions. When exercising without a mask, a large amount of mostly 0.3–0.5 μm sized particles was emitted in addition to 0.5–1.0 and 1.0–5.0 µm particles. Larger particles with a size of >5 μm were almost at zero measured concentration. The increase in particles emission started almost immediately after the subject started training and continued to increase for about 30 s and continued to fluctuate until stopping the exercise. For the illustrated test subject, the emission rate of the 0.5–1.0 and 1.0–5.0 µm particles fluctuated steadily after reaching 75% of the maximum heart rate. The 0.3–0.5 µm particles, on the other hand, demonstrated two peaks at 93 and 98 s. When wearing the surgical mask, the emission of the smaller particles sized 0.3 to 5.0 µm was drastically decreased. Similar to exercising without a mask, the amount of the emitted particles with a size of >5 μm was near zero. When wearing the FFP2 mask, the measured particle emission was almost negligible for all particle sizes throughout the training period.

**Figure 4:**
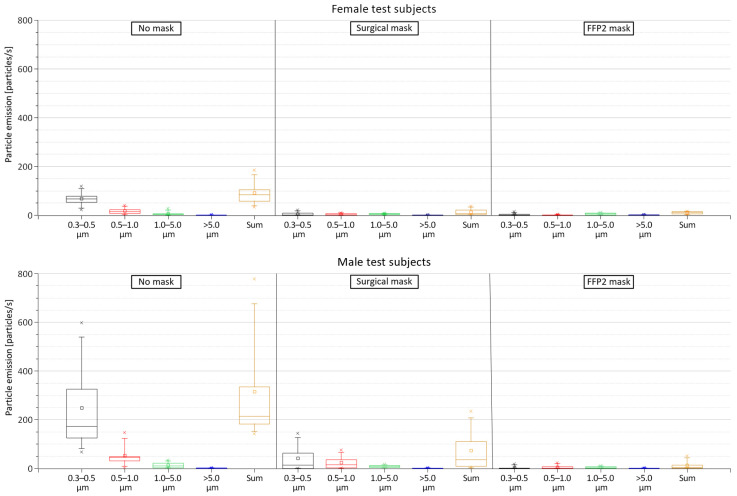
Particle emissions from the female and male test subjects when wearing no mask, surgical mask, and FFP2 mask.

The results indicated a strong variation in the emitted particles among the 12 test subjects with a sum of all emitted particle sizes when wearing no mask ranging from 32.92 to 777.75 particles/s (mean = 203.25 particles/s). Due to a large difference in particle emissions between the female and male subjects, Figure 4 shows the mean measured particles from these two groups separately. The box plots show that the female subjects emitted significantly fewer particles than the male test persons during all investigated mask conditions. With no mask, the average sum of all emitted particle sizes from the female subjects was 90.87 particles/s (standard deviation (SD) = 48.60 particles/s) compared to 315.62 particles/s (SD = 218.52 particles/s) from the male subjects. Thus, the male subjects had an emission rate more than three times higher than the female subjects. The surgical mask reduced the emission rates for all particle diameters from the female test subjects by an average of 81.7% (with an emission rate ranging from 2.14 to 36.32 particles/s). This reduction rate of the surgical masks was even higher for male subjects with an emission reduction of 92.5% (with an emission rate ranging from 0.29 to 234.07 particles/s). The same trend was observed with FFP2 masks, in which the reduction rate of all emitted particles was 85.3% and 97.3% for female and male subjects, respectively (with an emission rate of 1.41 to 15.99 particles/s and 0.92 to 51.05 particles/s for female and male subjects, respectively). The measured particle emission rates with FFP2 masks were, however, fairly low and can be attributed to measurement uncertainties related to the presence of particles in the room air with a concentration higher than the calculated average background concentration.

**Figure 5:**
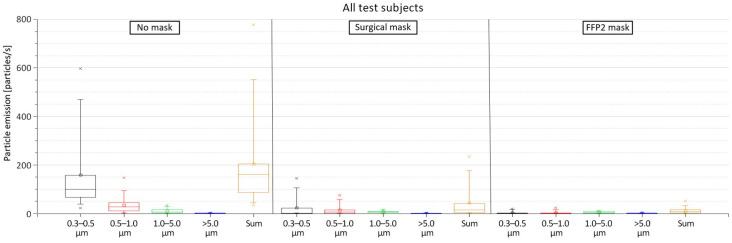
Particle emissions from all test subjects when wearing no mask, surgical mask, and FFP2 mask.

Figure 5 shows the measured particles emission from all 12 test subjects. The results indicate that the emission of particles with the size of 0.3–0.5 μm is always the highest, especially in the case of wearing no mask, in which the emission rate of 0.3–0.5 µm particles ranged from 21.79 to 587.20 particles/s (mean = 158.14 particles/s). This accounts for an average of 77.8% of the emitted particles. In addition to having the highest emission rate, the 0.3–0.5 µm particles showed the largest variation in comparison to the other particle sizes (SD = 158.70 particles/s). The second largest emitted particle size was 0.5–1.0 µm with a mean of 34.53 particles/s (17% of the emitted particles), followed by the particles sized 1.0–5.0 µm with a mean of 10.24 particles/s (5% of the emitted particles) and, lastly, the particles sized >5.0 µm with a mean of 0.34 particles/s (0.2% of the emitted particles).

When wearing a surgical mask, the sum of all emitted particle sizes was reduced by 87.1%; the sum of all emitted particle sizes ranged from 0.29 to 234.07 particles/s. Despite the significant reduction in their emission rate when wearing the surgical mask, the smaller particles with the size of 0.3–0.5 µm still had the highest emission with an average of 22.87 particle/s and accounted for an average 53% of the emitted particles. The other measured particle sizes had an average emission rate of 13.66, 6.30, and 0.36 particles/s for the particle size ranges of 0.5–1.0, 1.0–5.0, and >5.0 µm, respectively. This corresponded to an average of 31.6%, 14.6%, and 0.8% of the emitted particles, respectively.

**Figure A1:**
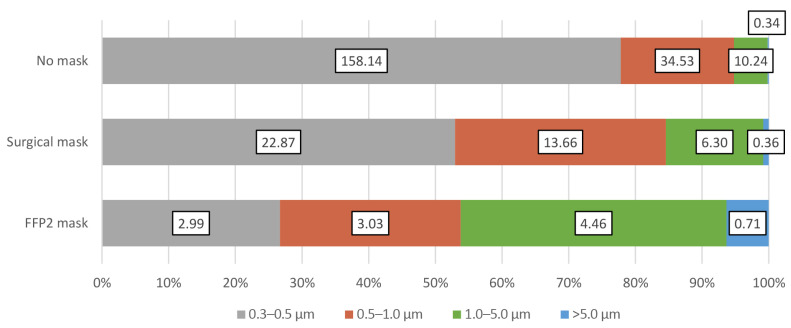
The mean particle emission rate [particles/s] and the mean size distributions of the emitted particles from all test subjects when wearing no mask, surgical mask, and FFP2 mask.

**Figure A2:**
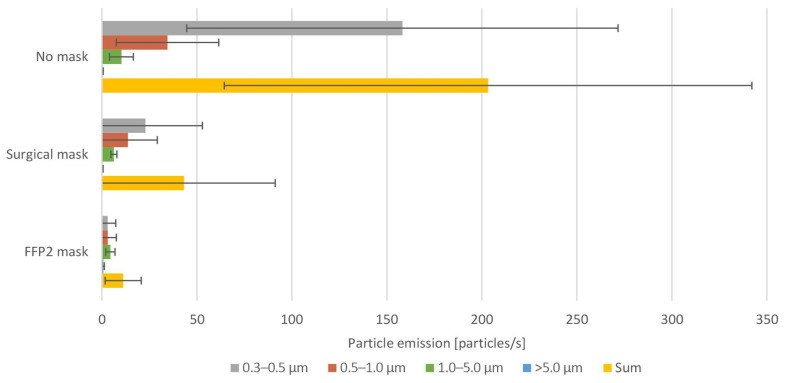
The mean emission rate of the exhaled particles and their confidence intervals (α = 0.05) from all test subjects when wearing no mask, surgical mask, and FFP2 mask.

When wearing an FFP2 mask, the accumulated particle emission rate was even further reduced with a reduction average of 91.3% compared to wearing no mask. In this case, the sum of emitted particles of all sizes ranged from 0.92 to 51.05 particles/s. This reduction was, in particular, due to the decrease in the emission rates of the smaller 0.3–0.5 µm particle size range. However, for the larger particle sizes, no significant difference was apparent in comparison to the surgical mask. However, when wearing the FFP2 mask, no big differences in the emission of the various particle sizes can be detected. The size distribution of the different measured particle sizes is presented in Figure A1 in the Appendix A; Figure A2 shows the mean emission rate of the exhaled particles and their confidence intervals (α = 0.05). The exact values of the particle emission as well as the reduction rate when wearing the masks are presented in Table A1 in the Appendix A.

### 3.2. Airflow Visualization

**Figure 6:**
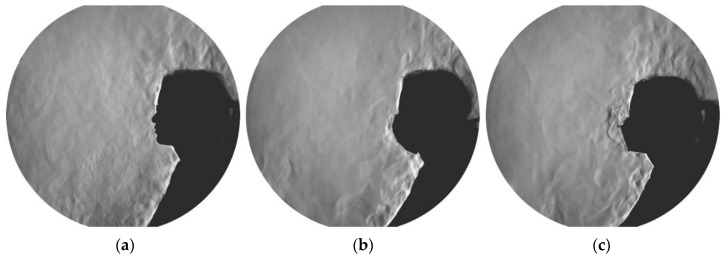
Schlieren images of a female test subject when wearing no mask (**a**), surgical mask (**b**), and FFP2 mask (**c**). The corresponding videos of these images are available in the Supplementary Materials (Videos S1, S2, and S3).

Figure 6 presents an example of the schlieren visualization images of a 24-year-old female test subject during the three investigated mask conditions. In the no mask scenarios, the exhaled air extended beyond the convective airflow surrounding the subjects and exceeded the measurement area of the schlieren mirror (Video S1 in the Supplementary Materials). Conversely, when wearing a surgical mask, the exhaled cloud did not exceed the convective flow above the extended arms and thighs of the test subject; the exhaled air was, thus, lifted and merged with the flow of the thermal plume above the body (Video S2 in the Supplementary Materials). The FFP2 mask constituted a tighter obstacle in the path of the exhaled jet. Thus, the exhaled cloud did not exceed the convective flow directly in front of the face and was rapidly lifted upwards (Video S3 in the Supplementary Materials). The airflow visualizations of the exhaled air emitted by an exemplary 40-year-old male test subject can be found in the Supplementary Materials (Videos S4–S6). Similar to the observation made with the female test subject, the exhaled airflow from the male subject exceeded the measurement range of the mirror in the case of no mask. The released cloud from the surgical mask was larger in the case of the male test subject compared to the female test subject. Yet, the size of the released exhalation cloud from the FFP2 mask was comparable in the cases of the female and male test subjects.

**Figure 7:**
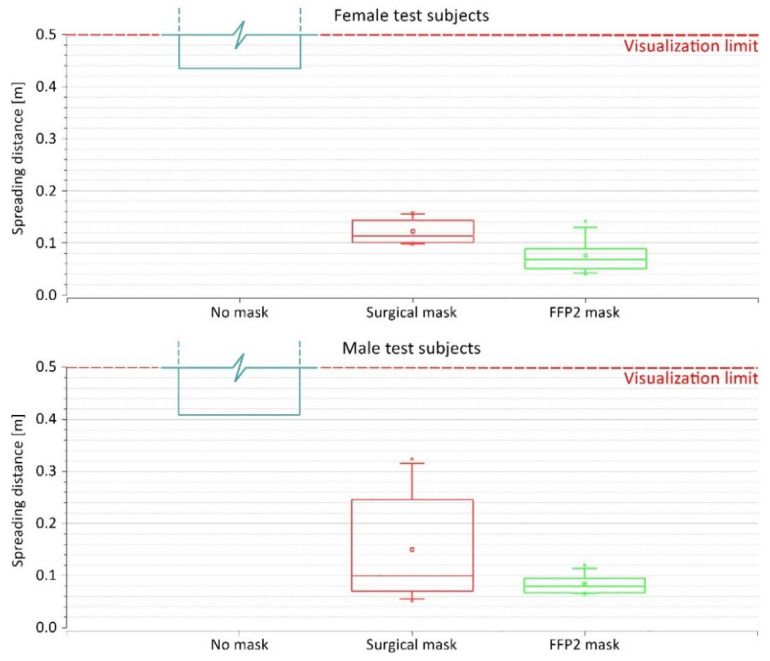
The spreading distance of exhaled air from the female and male test subjects when wearing no mask, surgical mask, and FFP2 mask.

**Figure 8:**
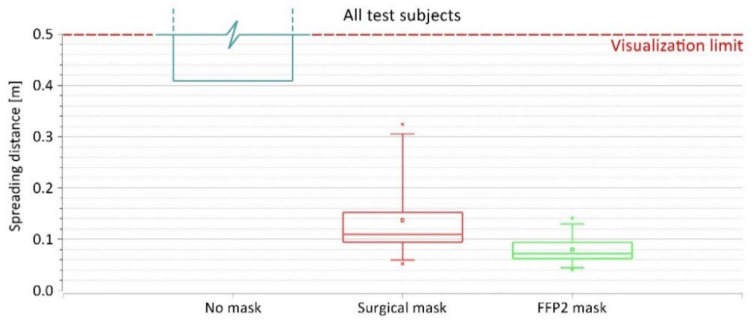
The spreading distance of exhaled air from all test subjects when wearing no mask, surgical mask, and FFP2 mask.

Aside from the qualitative assessments, the schlieren images were used to quantify the spreading distance of the exhaled air. This was manageable in the cases of surgical and FFP2 masks since the wavefront of the exhaled cloud was distinguishable and was always within the range of the measurement area of the mirror. However, in the case of no mask, the exhaled jet often exceeded the mirror visualization field. In such cases, an extrapolation of the spreading distances outside the mirror area can be done using the reduction of the expired air velocity [32]. However, in the present study, this was not possible due to the constant physical movement of the subjects and the thermal plume from the subjects’ lower bodies, which made the wavefront of the expired air often indistinguishable in the schlieren recordings. Therefore, in Figure 7 and Figure 8, the no mask cases only show the portion of the subjects that generated a distinguishable wavefront within the mirror area. The rest fell beyond the visualization limit of the mirror, which is marked with a red dotted line in the diagrams. As a result, for the no mask cases, the percentiles, the median, and the mean values were not calculated and not depicted in the boxplot diagrams.

Figure 7 shows the quantitative evaluation of the spreading distance of the exhaled plume from the female and male subjects. The patterns shown in this diagram align with the qualitative observations made from the schlieren images shown in Figure 6 and Videos S1–S6 in the Supplementary Materials. For both female and male subjects, wearing the face mask (regardless of the type) provided a significant reduction of the exhalation spreading distance compared to having no mask, in which the spreading distances exceeded the visualization field (ca. 0.5 m from the face, depending on the positions of the head in relation to the edge of the mirror). With female test subjects, the spreading distance when wearing a surgical mask ranged between 0.1 and 0.16 m with an average of 0.12 m. When wearing an FFP2 mask instead, the spreading range was reduced to 0.04 to 0.14 m with an average of 0.08 m. Male test subjects, on the other hand, demonstrated larger spreading distances when wearing face masks. With the surgical mask, the spreading distance of exhaled air ranged between 0.05 and 0.32 m with an average of 0.15 m. Similar to the pattern observed with female test subjects, the FFP2 mask reduced the spreading distance with male subjects as well. In this case, the spreading distance ranged between 0.06 and 0.12 m with an average of 0.08 m. Additionally, the results indicated a narrower variety of spreading distances of female subjects when wearing a surgical mask compared to the male subjects (SD = 0.03 and 0.12 m for female and male subjects, respectively). On the contrary, the variety of spreading distances when wearing an FFP2 mask was higher with female subjects compared to male subjects (SD = 0.04 and 0.02 m for female and male subjects, respectively). These patterns stem most likely from interindividual differences and different fitting of the masks related to the geometry of the face.

When considering all test subjects as one group, similar patterns related to the spreading distances can be observed (Figure 8). In this case, the larger variety of spreading distances occurred when wearing the surgical mask, with the distance ranging between 0.05 and 0.32 m, average = 0.14 m, and SD = 0.08 m. With the FFP2 mask, the spreading distance from all test subjects ranged between 0.04 and 0.14 m with an average of 0.08 m and an SD of 0.03 m.

### 3.3. User Satisfaction Surveys

Results regarding user satisfaction data with respect to RPE, RPD, thirst sensation, thermal sensation, and fatigue level are presented in Table 1. Solely RPD showed a statistically significant difference between the three mask conditions. Post-hoc tests with pairwise comparisons show that the mean RPD score was significantly different between no mask and FFP2 mask (p_adj_ = 0.042), but not between the no mask and the surgical mask (p_adj_ = 0.54) and the surgical mask and the FFP2 mask (p_adj_ = 0.13).

The results of the four assessed self-reported complaints are presented in Table 2. None of the participants reported any headaches or coughing. One participant each reported a short occurrence of dizziness with the surgical and FFP2 masks. Facial sweating was reported by one-fourth to one-third of the participants depending on the mask condition. Further analysis revealed that facial sweating was much more related to the repetition than the mask condition. Independent of the mask condition, no participant reported facial sweating after the first repetition, while four and five participants reported facial sweating after the second and third repetition, respectively.

## 4. Discussion and Conclusions

Various medical mask types are commonly used for personal protection and to limit the spreading of potentially contagious aerosol particles, e.g., SARS-CoV-2 or tuberculosis [1,42]. To verify the impact of this protective gear, this study analyzed the influence of frequently used masks, namely surgical and FFP2 masks, on aerosol particle emission and the spread of expiratory air during physical exercise. In particular, the study focused on aerosol particles smaller than 5 µm which almost indefinitely remain airborne in enclosed rooms and are prone in the lower respiratory tract of humans [4,5]. The hypothesis behind the study is that face masks can establish a significant decrease in particle count and exhaled air spreading during physical exercise compared to wearing no mask.

The presented results indicated an immediate increase in aerosol particle emission at the beginning of physical exercise reaching up to 777.75 particles/s (mean = 203.25 particles/s). In line with our findings, Mutsch et al. [21] demonstrated a 132-fold particle emission increase at maximal exercise; Wilson et al. [43] reported a 58-fold increase of aerosol particles during submaximal exercise. In the present study, the released aerosol plumes were analyzed containing mostly particles sized 0.3–0.5 µm in addition to a lower count of 0.5–1.0 µm sized particles. Interestingly, particles with a threshold of >5 µm were almost absent in all measurements, even with the absence of any kind of filtering mask. Similarly, Mutsch et al. [21] revealed a mean particle size of 0.46 ± 0.05 μm in the exhaled air, which coincides with the presented measurements. Additionally, the present findings illustrate a distinct variation in the overall particle emission, with a more than threefold higher emission rate from the male test subjects. Moreover, the size of the emitted particles differs between the male and female subjects in addition to a more or less expected interindividual variation. Although we assume that a greater aerosol production at higher levels of physical activity is likely to be a result of increased shear forces in the upper and lower airways [3,44] and correlated to increasing tidal and minute volumes, our setup did not yield any data proving our hypothesis.

This study demonstrates a significant reduction of all emitted particles when wearing a surgical mask by up to 87.1%. This observation supports our hypothesis that medical masks decrease the spreading rate and have a significant impact on personal protection. Nevertheless, the smallest particles of 0.3–0.5 µm were accountable at an average of 53% of the emitted particles when wearing a surgical mask. The FFP2 mask was able to reduce the overall particle emission by up to 91.3% compared to wearing no masks. This reduction was particularly due to the reduction of the critical 0.3–0.5 µm particles, indicating the superiority of the FFP2 masks for the prevention of infection risk. Yet, for the larger particles, no remarkable decrease was detected compared to the surgical mask. We observed a variation in reduction rates depending on the biological sex of the tested subjects. The reduction of exhaled particles when wearing a surgical mask was averagely 10.8% higher with male test subjects compared to female subjects. This value was increased to 12.0% when wearing an FFP2 mask instead of the surgical mask (Figure 4). In this context, we must emphasize that the fabric quality of the mask as well as the correct size and fitting must be ensured to provide the best possible protection for both the mask user and bystander [45,46]. In our study, the correct fitting of the masks was instructed, demonstrated, checked, and finally supervised by a physician. Nevertheless, air leakage might occur, primarily close to the nose and the chin, which are known as weak spots [45]. This can be seen in some of the schlieren imaging videos, e.g., Video S5 in the Supplementary Materials. Although Oestenstad et al. [46,47,48] have shown that the location of leaks on subjects wearing half-mask respirators depends highly on facial dimensions, we found no data to explain the difference between the reduction rates. A correlation of our schlieren visualization results in comparison to particle measurements was enjoined due to the different methods of measurement and should be performed in further studies to illustrate the effectiveness and limitations of these medical masks in more detail.

Several studies involving surgical masks and masks with a higher protection class (such as FFP2 and KN95) have shown their effectiveness in reducing the amount of exhaled particles during breathing, talking, and coughing by up to 90% and above [49,50,51]. This makes them suitable for reducing pathogen-containing aerosol plumes and decreasing the risk of cross-infection in daily life [52,53]. We were able to show that surgical masks and FFP2 masks are also suitable for physical activities significantly reducing exhaled particles (Figure 4). Unfortunately, we could not determine the exact spreading distance of the exhaled plume while wearing no mask due to technical limitations. However, our schlieren visualizations clearly showed a significant reduction of the spreading distance of the exhaled air when wearing a surgical mask or FFP2 mask (Videos S2, S3, S5, and S6 in the Supplementary Materials). The sealing effect of face masks for respiratory jets has recently been demonstrated, proving that any kind of face covering alters the trajectory and influences the travel distance of the exhaled air [54,55]. Using schlieren visualizations, Kerl et al. [31] proved that wearing a surgical mask as well as an FFP2 mask results in a diversion of the exhaled air and an uplifting effect by the thermal plume of the human body under resting conditions and while coughing. This observation was confirmed by our results (Figure 6). Furthermore, we demonstrated that wearing a medical face mask, independent of the type, restricts the exhaled air within the borders of the test environment (the cycle ergometer in this case), rendering them sufficient protection gear even under physical stress.

The presented data emphasizes that increasing physical exercise leads to increased aerosol emissions (Figure 3). Moreover, the data showed that both investigated masks provide a sufficient reduction in particle emission and the spread of the exhaled air plume, with a slight advantage of the FFP2 mask (Figure 5). Since indoor group exercises, such as in gym facilities, have been identified in the past as potential outbreak clusters for airborne infections [56], we strongly recommend wearing at least a surgical mask in combination with current hygiene standards such as social distancing and proper ventilation during a respiratory disease pandemic. In particular, we found no significant benefit of the FFP2 mask in comparison to the surgical mask in reducing exhaled particles under physical stress. However, FFP2 masks provide better filtering for both, the wearer in a pathogen-laden environment and of course the bystander and, thus, better protection for the wearer [57,58].

When wearing an FFP2 mask, Cabanillas-Barea et al. [59] found no significant impact on heart rate, oxygenation, or inspiratory tone accessory muscles in a 6 min walking test but showed an influence on perceived dyspnea. The present study indicated a significant difference between the perceived dyspnea while wearing an FFP2 mask in comparison to wearing no mask, but not between a surgical mask and no mask. This could lead to a reduced acceptance of FFP2 masks during physical exercise. Fikenzer et al. [60] reported a stronger impairment of ventilation, cardiopulmonary exercise capacity, and comfort when FFP2 masks during physical exercise compared to surgical masks. This must also be considered in whether to rely on an FFP2 mask or a surgical mask. In comparison to Zheng et al. [23], RPE and thermal sensation did not differ due to face mask use in our study. Our observation of increased RPD with face mask usage is in line with their findings. Yoshihara et al. [25] found RPE, thermal sensation, fatigue level, and overall breathing discomfort to be higher after 30 and 60 min exposures, while they observed no difference between mask conditions in RPE, thermal sensation, thirst sensation, and fatigue level. Solely the breathing comfort was significantly affected during the investigations with face masks. These findings are overall in line with those obtained in this study, in which no differences were found in RPE, thermal sensation, thirst sensation, and fatigue level between the three mask conditions after short-term sports activities. Only RPD, which may be comparable to breathing discomfort used by Yoshihara et al. [25], showed significant differences between mask conditions. In contrast to İpek et al. [24], who looked at non-exercising healthcare workers, no systematic differences were found in either of the four self-reported physical and psychological symptoms. An explanation is likely the length of wearing masks, as this was 1 to 4 h in their study, while a few minutes in the current study.

Overall, with respect to user satisfaction, the small sample size has to be regarded as a clear limitation of the data set. In addition, due to conducting the surveys simultaneously with the airflow visualizations, all questions were asked only after the exposure and not during the exposure as recommended, which added additional noise to the data. Furthermore, the exposure periods were likely too short to exert high influences on aspects related to user satisfaction and self-reported symptoms. The relatively small, heterogenous group of participants also constituted a limitation for assessing the particle emissions and spread of exhaled air since the participants had different levels of physical fitness, including smokers as well as non-smokers. This resulted in inhomogeneous exercise lengths since keeping the threshold of 75% maximal heart rate under exercise for 30 s was the aim. Although no medical emergencies arose, it was necessary to abort one measurement for safety reasons. For future studies, it appears reasonable to include a higher number of participants and divide them into different sub-groups, e.g., smokers and non-smokers, and by physical activity level. In addition, a baseline of exhaled particles prior to starting the exercise should be gathered. Thereby, it might be easier to explain interindividual differences. Moreover, a longer period of physical exercise should be evaluated. Furthermore, the assessments were conducted on a stationary exercise device, a cycle ergometer. This resulted in only little movements of the test subjects and a relatively homogeneous convective flow around them. Other physical activities, such as training on a treadmill ergometer or weightlifting may result in a different aerosol spreading. Additionally, the measurements were performed in a grey room, which limited the analysis and evaluation. Background aerosol concentration could not be completely eliminated, leading to measurement uncertainties. Hence, further research is needed to elaborate on the minimal differences between surgical and FFP2 masks.

In conclusion, we observed a significant increase in exhaled aerosol particles during physical exercise. Our results show that the use of surgical and FFP2 masks is suitable to reduce particle spreading with a nearly tenfold reduction benefit of the FFP2 mask over the surgical mask in the emission of the longer residing small exhaled particles (0.3 to 0.5 µm). We were able to show clear benefits to the wearer and others in the same room from wearing a mask of any protection class during exercise indoors, with a marked superiority of the FFP2 mask over the surgical mask. Both mask types decrease the travel distance of exhaled air under physical exercise, thus alleviating the risk of infection. The reported findings can be used as a basis to develop safety recommendations for indoor group exercises for the containment of airborne infections.

## Figures and Tables

**Table 1:** Means, standard deviations, and test statistics of repeated-measures ANOVA.

	Mean and Standard Deviation			ANOVA Test Statistics		
	No Mask	Surgical Mask	FFP2 Mask	F(dfn/dfd)	*p*	Eta^2^_tot_
Perceived exertion (RPE)	12.2 ± 1.96	12.9 ± 2.11	13.2 ± 2.22	2.8(2/22)	0.08	0.041
Perceived dyspnea (RPD)	10.8 ± 1.27	11.6 ± 1.9	13.1 ± 2.8	6.1(2/22)	**0.008**	0.18
Thirst sensation	1.42 ± 0.5	1.67 ± 0.9	1.33 ± 0.9	1.3(2/22)	0.28	0.03
Thermal sensation	6 ± 1	6.3 ± 1.3	6.4 ± 1.1	1.4(2/22)	0.27	0.02
Fatigue level	3.4 ± 1.3	3.8 ± 1.2	3.8 ± 1.8	0.6(1.2/13.2)	0.49	0.02

**Table 2:** Frequencies and response time of physical and psychological symptoms by mask condition.

Physical and Psychological Symptoms	No		1 min		2 min		5 min		>5 min
	*n*	%	*n*	%	*n*	%	*n*	%	*n*
Headache									
No mask	12	100	-		-		-		-
Surgical mask	12	100	-		-		-		-
FFP2 mask	12	100	-		-		-		-
Dizziness									
No mask	12	100	-		-		-		-
Surgical mask	11	91.7	1	8.3	-		-		-
FFP2 mask	11	91.7	1	8.3	-		-		-
Facial sweating									
No mask	9	75	3	25	-		-		-
Surgical mask	8	66.7	2	16.7	2	16.7	-		-
FFP2 mask	10	83.3	2	16.7	-		-		-
Coughing									
No mask	12	100	-		-		-		-
Surgical mask	12	100	-		-		-		-
FFP2 mask	12	100	-		-		-		-

## Data Availability

The research data of this study can be made available by the corresponding authors upon request.

## Supplementary Materials

The following supporting information can be downloaded at: https://www.mdpi.com/article/10.3390/jcm12041300/s1, Video S1: Schlieren imaging video of the expired air from a female participant with no mask; Video S2: Schlieren imaging video of the expired air from a female participant with a surgical mask; Video S3: Schlieren imaging video of the expired air from a female participant with an FFP2 mask; Video S4: Schlieren imaging video of the expired air from a male participant with no mask; Video S5: Schlieren imaging video of the expired air from a male participant with a surgical mask; Video S6: Schlieren imaging video of the expired air from a male participant with an FFP2 mask.

## Appendix A

**Table A1:** The minimum, maximum, mean, median, and standard deviation of the measured particles during the investigated mask conditions and the reduction efficiency of the tested masks.

	Female Subjects					Male Subjects					All Subjects				
	0.3–0.5 µm	0.5–1.0 µm	1.0–5.0 µm	>5.0 µm	Sum	0.3–0.5 µm	0.5–1.0 µm	1.0–5.0 µm	>5.0 µm	Sum	0.3–0.5 µm	0.5–1.0 µm	1.0–5.0 µm	>5.0 µm	Sum
No mask: Measured values [particles/s]															
Min	21.79	0.00	0.00	0.00	32.92	67.56	1.44	1.04	0.00	141.83	21.79	0.00	0.00	0.00	32.92
Max	117.70	39.36	26.24	0.95	184.24	597.20	146.50	33.44	1.31	777.75	597.20	146.50	33.44	1.31	777.75
Mean	67.08	16.57	7.03	0.18	90.87	249.20	52.48	13.45	0.49	315.62	158.14	34.53	10.24	0.34	203.25
Median	65.75	14.31	4.12	0.00	83.93	172.72	45.19	10.14	0.38	213.87	99.17	26.09	4.98	0.06	160.72
SD	29.08	12.74	8.77	0.35	48.60	181.51	45.08	11.65	0.49	218.52	158.70	37.68	10.80	0.45	194.13
Surgical mask: Measured values [particles/s]															
Min	0.00	0.00	2.05	0.00	2.14	0.00	0.00	0.00	0.01	0.29	0.00	0.00	0.00	0.00	0.29
Max	20.46	9.18	7.92	0.59	36.32	143.78	74.20	15.38	1.05	234.07	143.78	74.20	15.38	1.05	234.07
Mean	5.05	3.04	4.80	0.27	13.16	40.70	24.29	7.79	0.46	73.23	22.87	13.66	6.30	0.36	43.19
Median	0.00	0.89	4.91	0.27	6.30	13.16	14.95	7.21	0.34	35.65	0.00	4.48	6.25	0.34	13.46
SD	7.77	3.75	2.18	0.25	13.07	53.10	26.35	5.06	0.34	83.87	41.92	21.61	4.17	0.31	67.12
Surgical mask: Emission reduction compared to no mask [%]															
Min	67.8	29.2	47.1	53.9	53.8	87.6	71.6	31.8	17.1	83.5	67.8	29.2	31.8	17.1	53.8
Max	100.0	99.6	69.8	53.9	98.0	100.0	100.0	100.0	99.2	99.9	100.0	100.0	100.0	99.2	99.9
Mean	93.2	71.2	58.4	53.9	81.7	96.9	89.5	62.7	62.0	92.5	94.7	81.4	61.5	60.0	87.1
Median	100.0	78.1	58.4	53.9	86.2	100.0	100.0	62.5	69.7	95.1	100.0	81.5	62.5	61.8	90.9
SD	11.8	25.9	11.4	0.0	16.1	5.4	12.9	23.2	33.9	6.3	9.9	21.8	20.7	29.6	13.3
FFP2 mask: Measured values [particles/s]															
Min	0.00	0.00	1.32	0.00	1.41	0.00	0.00	0.41	0.00	0.92	0.00	0.00	0.41	0.00	0.92
Max	14.10	2.88	9.59	1.95	15.59	18.09	22.55	10.23	1.69	51.05	18.09	22.55	10.23	1.95	51.05
Mean	2.96	0.64	5.23	0.90	9.73	3.01	5.43	3.70	0.52	12.67	2.99	3.03	4.46	0.71	11.20
Median	0.00	0.00	4.89	0.77	11.14	0.00	0.63	1.78	0.37	3.40	0.00	0.11	2.73	0.37	7.09
SD	5.16	1.06	3.41	0.83	5.14	6.74	8.26	3.64	0.55	17.90	6.00	6.36	3.61	0.73	13.25
FFP2 mask: Emission reduction compared to no mask [%]															
Min	77.8	92.7	38.3	29.2	71.6	97.0	84.6	69.4	59.7	93.4	77.8	84.6	38.3	29.2	71.6
Max	100.0	100.0	63.5	29.2	98.7	100.0	100.0	93.5	100.0	99.6	100.0	100.0	93.5	100.0	99.6
Mean	95.1	98.5	50.9	29.2	85.3	99.5	96.3	83.3	76.8	97.3	97.3	97.4	68.9	64.9	91.3
Median	100.0	100.0	50.9	29.2	86.2	100.0	99.2	83.3	70.8	98.0	100.0	100.0	69.4	65.2	95.1
SD	8.2	2.9	12.6	0.0	10.4	1.1	5.9	8.1	17.0	2.2	6.2	4.8	19.1	25.3	9.6
